# Association Between Integrated Treatment Services and Access to Detoxification and MOUD Across US Treatment Facilities

**DOI:** 10.1007/s11414-026-10003-9

**Published:** 2026-06-08

**Authors:** Fares Qeadan, Falak Choudry, Rose Thornquist

**Affiliations:** Parkinson School of Health Sciences and Public Health, Loyola University Chicago, Maywood, IL USA

## Abstract

Integrated treatment services for co-occurring mental health and substance use disorders are recommended best practice, yet their relationship with facility-level provision of detoxification and medications for opioid use disorder (MOUDs) is not well understood. The researchers analyzed 2021–2023 National Substance Use and Mental Health Services Survey (N-SUMHSS) data from 49,623 facilities offering any substance use services. Multivariable logistic regression models examined associations between integrated services and availability of detoxification and MOUD, adjusting for facility characteristics and Chronic Care Model elements. Primary treatment focus (substance use, mental health, mixed) was evaluated as an effect modifier. Overall, 61.2% of facilities offered integrated services, 21.2% provided detoxification, and 56.0% provided MOUD. Among substance use–focused facilities, integrated services were associated with higher odds of offering detox (adjusted odds ratio [aOR] = 1.27, 95% CI 1.17–1.37) and MOUD (aOR = 1.44, 95% CI 1.34–1.56). Among mixed-focus facilities, integrated services were associated with higher odds of MOUD (aOR = 1.39, 95% CI 1.28–1.51) but not detoxification. Among mental health–focused facilities, integrated services were associated with lower odds of detoxification (aOR = 0.34, 95% CI 0.22–0.53) and did not predict MOUD availability. Facility type, ownership, and Chronic Care Model elements, including delivery system redesign, self-management support, and clinical information systems, were strong independent predictors of both outcomes. Integrated services may be associated with greater access to detoxification and MOUD in substance use–focused settings but appear insufficient within mental health–focused facilities; efforts to strengthen integrated care should account for facility focus and infrastructure capacity.

**Supplementary Information:**

The online version contains supplementary material available at https://doi.org/10.1007/s11414-026-10003-9.

## Introduction

Research estimates that 23.1% of the US adult population (i.e., 59.3 million people) lives with a mental illness.^1^ In 2020, around 40.3 million people in the USA had a substance use disorder (SUD).^2^ According to the 2022 National Survey on Drug Use and Health, around 21.5 million adults face a co-occurring disorder.^3,4^ Among adults with opioid use disorder (OUD), specifically, the prevalence of characteristics associated with past-year mental illness was 64.3%.^5^ As many individuals experience mental illness in tandem with SUDs, a comprehensive treatment approach that targets both issues is crucial.^6^ However, only 24.5% of OUD patients with co-occurring mental illness reported receiving both mental health and substance use treatment in the past year,^5^ highlighting a significant gap in comprehensive treatment for patients with co-occurring disorders.

In response to the widespread relationship between substance use and mental health disorders, integrated treatment has emerged as a critical approach to addressing both conditions simultaneously through a coordinated treatment plan. It is known that an integrated treatment plan is more effective than treating mental illness or SUDs alone,^7–10^ but access to facilities that offer integrated treatment is an ongoing issue.^11–13^ A recent literature review has identified multiple system-level barriers to integrated treatment access, including a lack of leadership support, policy constraints, and limited training, funds, community resources, and expert personnel.^14^

An equally urgent yet understudied concern is whether integrated services provide comprehensive, evidence-based care for SUDs, such as detoxification services and medications for OUD (MOUDs). Medical detoxification treatment, commonly referred to as detox, safely stabilizes individuals experiencing withdrawal symptoms and can be used for several types of SUDs, including OUD. While detox can be an effective starting point for OUD treatment, without follow-up treatment, such as MOUD, it is associated with high relapse rates and poor outcomes.^15–17^ MOUD includes specific medications such as methadone and buprenorphine that help OUD patients reduce cravings and prevent relapse.^18,19^ However, a 2020 retrospective study found that only 15.8% of OUD patients received detox and 14.9% received MOUD,^20^ suggesting significant access barriers. Barriers to detox include time constraints for patients and limited availability of long-term inpatient resources.^21^ Many facilities also lack the necessary support to establish a continuation of care in the transition between inpatient and outpatient settings.^21^ Specific barriers to MOUD include increased stigma, insufficient training, limited specialists, and a lack of MOUD prescribers, particularly in rural areas.^22–24^

Despite the importance of integrated services, empirical evidence on how the availability of such services affects the provision of key treatments like detox and MOUD is limited, particularly across different types of facilities and geographic regions. A study examining dual diagnosis treatment for both mental health and substance use between 2010 and 2018 discovered an increase in the number of facilities offering mental health services. However, the proportion of facilities that offered dual diagnosis integrated treatment has decreased over the same period of time.^25^ What remains unclear is how the availability of integrated services influences access to evidence-based SUD treatments, including detox and MOUD, across different contexts. Given the decline in integrated treatment and the implication that individuals with co-occurring disorders may not be receiving the coordinated care they need, including detox and MOUD, further research is needed to better understand the relationship between integrated treatment and evidence-based SUD treatment methods, as well as the characteristics of the facilities that offer them.

This explanatory study aims to fill that gap by examining whether facilities that offer integrated services are more likely to provide comprehensive care, including detox and MOUD, compared to facilities that do not offer integrated services. The Donabedian model conceptualizes care quality as a function of structural characteristics and care processes that shape outcomes.^26^ In this framework, facilities with integrated services may have greater resources and coordination to deliver comprehensive SUD treatment. As such, the current study hypothesizes that facilities that offer integrated services will be more likely to provide detox and MOUD. Understanding these relationships can help policymakers and healthcare providers optimize resource allocation and service offerings to improve treatment outcomes for individuals with co-occurring disorders.

## Methods

### Data source, sample, and design

Data were utilized from the 2021–2023 National Substance Use and Mental Health Services Survey (N-SUMHSS), a nationally representative voluntary survey administered by the Substance Abuse and Mental Health Services Administration (SAMHSA). This survey collects information on the services provided by public and private mental health and substance use facilities across the USA. Before 2021, data were collected using the National Survey of Substance Abuse Treatment Services (N-SSATS) and the National Mental Health Services Survey (N-MHSS). These two surveys were merged in 2021 to form the N-SUMHSS. The N-SUMHSS collects three types of information from facilities, including the characteristics of individual facilities, client count information (by service type and number of beds designated for treatment), and general information (licensure, certification, or accreditation and facility website availability).^27^ In the 2021 N-SUMHSS survey, data were collected on 28,298 eligible substance use and/or mental health treatment facilities across the USA, its territories, and the District of Columbia.^28^ In the 2022 N-SUMHSS survey, data were collected on 25,065 eligible substance use and/or mental health treatment facilities.^29^ In the 2023 N-SUMHSS survey, data were collected on 25,148 eligible substance use and/or mental health facilities.^30^

Of the 92,494 facilities surveyed between 2021 and 2023 (including those found to be either closed or ineligible), the study population was restricted to all facilities that provided a definitive response for the integrated services survey question between 2021 and 2023 (*n* = 49,623). This removed facilities that logically skipped the question (*n* = 17,240), as well as those missing a response (*n* = 25,631). Facilities that were logically skipped included those that only provided mental health services. All findings, therefore, should be interpreted as among facilities offering substance use treatment. A definitive response to the integrated services question was required for inclusion as this was the primary exposure of the study and was included in all multivariable logistic regression models. In SAS, these models use complete case analysis by default. This study was reviewed by the Loyola University Chicago Institutional Review Board (LU# 219811) and determined to be exempt under 45 CFR 46.104(d)(4)(i) as secondary research using publicly available data.

### Outcomes and predictors

The study focused on two binary outcome variables: (1) whether the facility offers detoxification services and (2) whether the facility offers MOUD. The cleaned binary (yes/no) detoxification variable provided by N-SUMHSS was used for this study. Specifically, the N-SUMHSS item asked, “Which of the following substance use treatment services are offered by this facility at this location, that is, the location listed on the front cover?”, and facilities could mark “yes” or “no” for “Detoxification (medical withdrawal).” MOUD was measured from the item “How does this facility treat opioid use disorder?”. If facilities answered, “This facility prescribes naltrexone to treat opioid use disorder,” “This facility utilizes prescribers of buprenorphine to treat opioid use disorder,” or “This facility is a federally certified Opioid Treatment Program (OTP),” the facility was coded as offering MOUD.

The primary predictor in this study was whether the facility reported offering integrated treatment services for co-occurring mental health and substance use disorders (yes/no). Specifically, facilities were asked, “Which of the following substance use treatment services are offered by this facility at this location, that is, the location listed on the front cover?”, and facilities could mark “yes” or “no” for “Treatment for co-occurring substance use plus either serious mental illness (SMI) in adults and/or serious emotional disturbance (SED) in children.”

**Figure 1:**
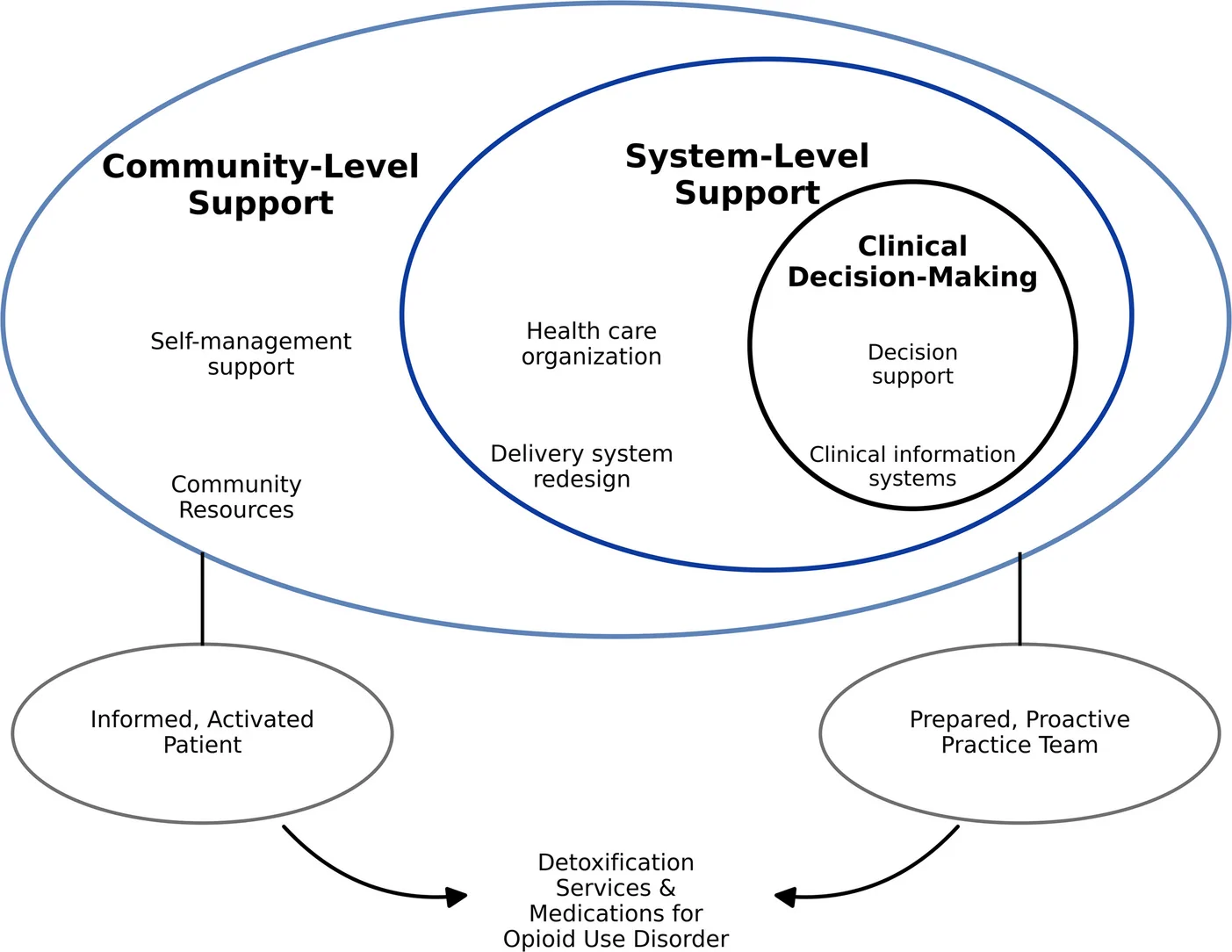
Elements of the Chronic Care Model: application to detoxification services and medications for opioid use disorder using the N-SUMHSS 2021–2023, inspired by Gee et al.^32^

Secondary predictors included categorical facility-level characteristics and system-level practices that may influence the availability of detoxification and MOUD services. Facility characteristics included facility type (i.e., inpatient only, outpatient only, residential only, mixed, and other), geographic location (i.e., Northeast, Southwest, West, Southeast, Midwest, and other jurisdictions), facility operation (i.e., public, private nonprofit, and private for-profit), and year (i.e., 2021, 2022, and 2023). System-level practices included binary proxy measures of elements from the Chronic Care Model (CCM) adapted from Watkins et al.^31^ Figure 1, inspired by Gee et al., visually presents the CCM.^32^ Elements included delivery system redesign (whether facilities used 12-step facilitation, brief intervention, contingency motivation incentives, trauma-related counseling, anger management, or matrix model for opioid use), self-management support (whether facilities provide patients with education about substance use and providing counseling to help them manage or overcome substance use issues), decision support (whether facilities provide patients with a professional consultant who assists in managing substance use issues), clinical information systems (whether facilities implement a complete medical history and physical examination), healthcare organization (whether facilities are licensed or certified by their state substance use agency), and community resources (whether facilities implemented recovery mentoring/peer support, self-help groups, housing assist, employment, assistance social services, a coach, case management, or a transitional discharge plan). Elements of the CCM, their application to detox and MOUD, and their proxy measure from N-SUMHSS are outlined in Supplemental Table 1.

Primary treatment focus as an effect modifier in the relationship between integrated services and outcomes was examined, assessing whether the impact of integrated services differs by the type of care the facility prioritizes. This variable showed whether a facility identified its main treatment focus as substance use, mental health, or mixed.

### Statistical analysis

Descriptive statistics were used to describe the facility-level characteristics and elements of the CCM overall and stratified by whether the facility used integrated services. Frequencies and percentages were calculated for all. The significance of predictors was tested with chi-square tests of independence.

The associations between integrated services and outcomes detox and MOUD were assessed through multivariable (i.e., multiple) logistic regression. Unadjusted odds ratios (ORs) and adjusted odds ratios (aORs) along with 95% confidence intervals (CIs) were calculated to represent these associations. Models were adjusted for all facility characteristics and elements of the CCM. Statistical interaction was used to explore whether facilities’ primary treatment focus was an effect modifier of the association between integrated services and outcomes. The associations between facility characteristics and outcomes detoxification services and MOUD were additionally assessed through multiple logistic regression. ORs and aORs along with 95% CIs were calculated to represent these associations. Complete case analysis was used for all analyses.

Goodness-of-fit (GOF) was assessed visually with Pearson and Deviance residuals and tested formally with Hosmer and Lemeshow’s test, Stukel’s test, and Osius and Rojek’s test. Influential observations were assessed with DFBETAS. The predictive ability of models was assessed with the area under the receiver operating characteristic (ROC) curve (AUC). The multicollinearity of predictors was assessed using a correlation matrix, and no significant multicollinearity was found. All hypothesis tests were two-sided with a significance level of 5%. SAS version 9.4 (SAS Institute, Inc.) was used for all analyses.

## Results

Table 1 provides the overall characteristics of US treatment facilities between 2021 and 2023. Of the 49,623 total facilities in the data sample, 30,389 (61.24%) provided integrated services for co-occurring mental health and substance use disorders. Among the total sample, most facilities were outpatient only (72.94%), incorporated a delivery system redesign (i.e., had evidence-based strategies for OUD treatment) (92.82%), and provided community resources (97.89%). While most (55.97%) facilities offered MOUD, only 21.23% offered detox. A higher proportion of outpatient only facilities was observed among those with integrated services than those without (74.95% vs. 69.75%), while a lower proportion was observed for residential only facilities (11.31% vs. 16.21%). A higher proportion of public (12.46% vs. 7.26%) and private nonprofit (52.65% vs. 44.51%) but a lower proportion of private for-profit (34.89% vs. 48.23%) facilities were found in treatment facilities with integrated services than without. A higher proportion of facilities that offered decision support (i.e., have expert consultation available to help clinicians adhere to evidence-based OUD treatment practices) was observed among those with integrated services than those without (32.83% vs. 21.97%) (Table 1).

**Table 1:** Overall characteristics of US treatment facilities: N-SUMHSS 2021–2023

		Integrated services^a^		
	Total *n*^b^ (%^c^)	No *n*^b^ (%^c^)	Yes *n*^b^ (%^c^)	*p* value^d^
Total^e^	49,623 (100)	19,234 (38.76^f^)	30,389 (61.24^f^)	
Facility type				< 0.0001
Inpatient only	920 (1.86)	384 (2.00)	536 (1.76)	
Outpatient only	36,154 (72.94)	13,381 (69.75)	22,773 (74.95)	
Residential only	6544 (13.20)	3109 (16.21)	3435 (11.31)	
Mixed	5106 (10.30)	1523 (7.94)	3583 (11.79)	
Other	843 (1.70)	786 (4.10)	57 (0.19)	
Geographic location				< 0.0001
Northeast	10,831 (21.83)	4546 (23.64)	6285 (20.68)	
Southwest	4235 (8.53)	1561 (8.12)	2674 (8.80)	
West	10,813 (21.79)	4487 (23.33)	6326 (20.82)	
Southeast	11,358 (22.89)	4080 (21.21)	7278 (23.95)	
Midwest	12,114 (24.41)	4432 (23.04)	7682 (25.28)	
Other jurisdictions^g^	272 (0.55)	128 (0.67)	144 (0.47)	
Facility operation				< 0.0001
Public	5175 (10.45)	1394 (7.26)	3781 (12.46)	
Private nonprofit	24,517 (49.49)	8543 (44.51)	15,974 (52.65)	
Private for-profit	19,844 (40.06)	9257 (48.23)	10,587 (34.89)	
Year				< 0.0001
2021	17,706 (35.68)	6758 (35.14)	10,948 (36.03)	
2022	17,071 (34.40)	6437 (33.47)	10,634 (34.99)	
2023	14,846 (29.92)	6039 (31.40)	8807 (28.98)	
Delivery system redesign^h^				0.4602
No	3361 (7.18)	1291 (7.30)	2070 (7.11)	
Yes	43,434 (92.82)	16,405 (92.70)	27,029 (92.89)	
Self-management support^h^				0.4919
No	1872 (3.81)	739 (3.88)	1133 (3.76)	
Yes	47,315 (96.19)	18305 (96.12)	29,010 (96.24)	
Decision support^h^				< 0.0001
No	34,946 (71.36)	14,760 (78.03)	20,186 (67.17)	
Yes	14,024 (28.64)	4156 (21.97)	9868 (32.83)	
Clinical information systems^h^				0.5166
No	29,460 (60.12)	11,353 (59.94)	18,107 (60.23)	
Yes	19,545 (39.88)	7589 (40.06)	11,956 (39.77)	
Healthcare organization^h^				< 0.0001
No	14,163 (29.40)	4859 (26.07)	9304 (31.50)	
Yes	34,013 (70.60)	13,782 (73.93)	20,231 (68.50)	
Community resources^h^				< 0.0001
No	1044 (2.11)	480 (2.51)	564 (1.86)	
Yes	48,416 (97.89)	18,675 (97.49)	29,741 (98.14)	
Primary treatment focus				< 0.0001
Substance use	20,375 (41.06)	14,117 (73.40)	6258 (20.59)	
Mental health	3004 (6.05)	640 (3.33)	2364 (7.78)	
Mixed	26,244 (52.89)	4477 (23.28)	21,767 (71.63)	
Detoxification services				0.0895
No	39,088 (78.77)	15,225 (79.16)	23,863 (78.53)	
Yes	10,533 (21.23)	4007 (20.84)	6526 (21.47)	
MOUD^i^				< 0.0001
No	21,682 (44.03)	8876 (46.60)	12,806 (42.41)	
Yes	27,561 (55.97)	10,171 (53.40)	17,390 (57.59)	

^a^Facility provides integrated services for co-occurring mental health and substance use disorders

^b^*n* = sample size; not all counts add up to total because of missing values

^c^Column percentage

^d^Chi-square test for independence

^e^Sample restricted to facilities with nonmissing values for integrated services; prevalences of integrated services may be inflated

^f^Row percentage

^g^This includes Puerto Rico, the Commonwealth of the Northern Mariana Islands, the Republic of Palau, and the US Virgin Islands

^h^ Element of the Chronic Care Model; construction of each element outlined in Supplemental Table 1

^i^Medications for opioid use disorder

Table 2 showcases the odds of detox and MOUD for integrated services by primary treatment focus as the association between integrated treatment services and both MOUD and detox depended on the facilities’ primary treatment focus. The associations between integrated treatment services and the outcomes detox and MOUD were both significant and positive for facilities whose primary treatment focus was substance use, with an aOR (95% CI) of 1.265 (1.167–1.371) for detox and 1.443 (1.339–1.555) for MOUD. Among mixed facilities, facilities with integrated services had 1.394 (1.284–1.513) times the odds of MOUD services than facilities without integrated services, but the association was not statistically significant for detoxification. Among mental health facilities, facilities with integrated services had 66.3% lower odds of detox than those without integrated services (0.337, 0.215–0.527), but the odds of MOUD did not differ by whether facilities had integrated services.

**Table 2:** Odds of detoxification services and MOUD for integrated services by primary treatment focus

	Detoxification services		MOUD^a^	
	aOR^b^ (95% CI)	AUC^c^	aOR^b^ (95% CI)	AUC^c^
Integrated services^d^ Yes vs. no				
Focus = substance use	**1.265 (1.167–1.371)**	0.825	**1.443 (1.339–1.555)**	0.801
Focus = mental health	**0.337 (0.215–0.527)**		0.847 (0.632–1.134)	
Focus = mixed	1.079 (0.975–1.194)		**1.394 (1.284-1.513)**	

Bold values indicate statistical significance at the *p* < 0.05 level

^a^Medications for opioid use disorder

^b^Adjusted odds ratio and 95% confidence intervals; adjusted for facility type, geographic region, facility operation, year, and all elements of the Chronic Care Model

^c^Area under the receiver operating characteristic curve; 0.7–0.8 is acceptable, 0.8–0.9 is excellent, and > 0.9 is outstanding

^d^Facility provides integrated services for co-occurring mental health and substance use disorders

Table 3 provides the odds of detox for facility characteristics. Among facility types, outpatient only (0.029, 0.024–0.036), residential only (0.086, 0.069–0.106), mixed (0.221, 0.178–0.273), and other (0.022, 0.014–0.034) facilities had lower odds of detoxification services than inpatient only facilities. Private nonprofit facilities had 33.9% lower odds of providing detox compared to public facilities (0.661, 0.602–0.727), while private for-profit facilities had 63.0% higher odds of providing detox compared to public facilities (1.630, 1.486–1.788). Facilities that provided delivery system redesign (i.e., had evidence-based strategies for OUD treatment) had 4.472 times the odds of providing detox compared to facilities that did not provide delivery system redesign (4.472, 3.722–5.374). Facilities that provided self-management support (i.e., have patients encouraged to become informed and competent self-managers of their OUD) had 85% higher odds of providing detox compared to those that did not (1.850, 1.466–2.334).

**Table 3:** Odds of detoxification services for facility characteristics

	Detoxification services^a^	
	OR^b^ (95% CI)	aOR^c^ (95% CI)
Facility type		
Inpatient only	Ref	Ref
Outpatient only	**0.033 (0.027–0.039)**	**0.029 (0.024–0.036)**
Residential only	0.094 (0.079–0.112)	**0.086 (0.069–0.106)**
Mixed	**0.281 (0.235–0.336)**	**0.221 (0.178–0.273)**
Other	**0.010 (0.007–0.015)**	**0.022 (0.014–0.034)**
Geographic location		
Northeast	Ref	Ref
Southwest	**1.449 (1.331–1.577)**	**1.426 (1.283–1.586)**
West	**1.444 (1.353–1.541)**	**1.624 (1.500–1.759)**
Southeast	**1.307 (1.224–1.395)**	**1.194 (1.102–1.295)**
Midwest	**0.930 (0.870–0.995)**	**1.181 (1.088–1.281)**
Other jurisdictions^d^	1.267 (0.949–1.692)	**0.658 (0.465–0.931)**
Facility operation		
Public	Ref	Ref
Private nonprofit	**0.672 (0.623–0.724)**	**0.661 (0.602–0.727)**
Private for-profit	**1.479 (1.375–1.592)**	**1.630 (1.486–1.788)**
Year		
2021	Ref	Ref
2022	0.908 (0.863–0.956)	**0.712 (0.668–0.758)**
2023	**0.878 (0.833–0.927)**	**0.619 (0.580–0.661)**
Delivery system redesign^e^		
No	Ref	Ref
Yes	**5.702 (4.864–6.685)**	**4.472 (3.722–5.374)**
Self-management support^e^		
No	Ref	Ref
Yes	**2.662 (2.276–3.113)**	**1.850 (1.466–2.334)**
Decision support^e^		
No	Ref	Ref
Yes	**1.339 (1.278–1.402)**	1.023 (0.965–1.085)
Clinical information systems^e^		
No	Ref	Ref
Yes	**5.782 (5.511–6.066)**	**4.079 (3.859–4.311)**
Healthcare organization^e^		
No	Ref	Ref
Yes	**0.921 (0.879–0.966)**	*0.948 (0.890–1.009)*
Community resources^e^		
No	Ref	Ref
Yes	**1.788 (1.493–2.141)**	0.948 (0.744–1.208)

Bold values indicate statistical significance at the *p* < 0.05 level. Italicized values indicate statistical significance at the 0.05 ≤ *p* < 0.10 level

^a^Facility provides detoxification services

^b^Odds ratio and 95% confidence intervals

^c^Adjusted odds ratio and 95% confidence intervals

^d^This includes Puerto Rico, the Commonwealth of the Northern Mariana Islands, the Republic of Palau, and the US Virgin Islands

^e^Element of the Chronic Care Model; construction of each element outlined in Supplemental Table 1

Table 4 provides the odds of MOUD for facility characteristics. Among facility types, residential only facilities had 34.4% lower odds of providing MOUD services compared to inpatient only facilities (0.656, 0.535–0.804). Private nonprofit facilities had 35.9% lower odds of providing MOUD compared to public facilities (0.641, 0.594–0.692), while private for-profit facilities had 8.9% lower odds of providing MOUD compared to public facilities (0.911, 0.842–0.986). Facilities that provided delivery system redesign had 9.883 times the odds of providing MOUD compared to facilities that did not provide delivery system redesign (9.883, 8.739–11.177). Facilities that provided self-management support had 2.027 times the odds of providing MOUD compared to facilities that did not (2.027, 1.761–2.334). Facilities that provided clinical information systems had 7.738 times the odds of providing MOUD compared to facilities that did not (7.738, 7.349–8.148).

**Table 4:** Odds of MOUD for facility characteristics

	MOUD services^a^	
	OR^b^ (95% CI)	aOR^c^ (95% CI)
Facility type		
Inpatient only	Ref	Ref
Outpatient only	0.408 (0.351–0.474)	0.887 (0.729–1.080)
Residential only	**0.392 (0.335–0.458)**	**0.656 (0.535–0.804)**
Mixed	**0.762 (0.649–0.895)**	0.988 (0.804–1.214)
Other	**0.108 (0.086–0.895)**	**0.489 (0.357–0.669)**
Geographic location		
Northeast	Ref	Ref
Southwest	0.424 (0.394–0.456)	**0.323 (0.295–0.354)**
West	**0.378 (0.357–0.400)**	**0.330 (0.308–0.353)**
Southeast	**0.564 (0.534–0.597)**	**0.480 (0.448–0.514)**
Midwest	0.423 (0.401–0.447)	**0.417 (0.391–0.445)**
Other jurisdictions^d^	**0.194 (0.149–0.251)**	**0.102 (0.075–0.139)**
Facility operation		
Public	Ref	Ref
Private nonprofit	**0.695 (0.653–0.738)**	**0.641 (0.594–0.692)**
Private for-profit	**1.155 (1.085–1.230)**	**0.911 (0.842–0.986)**
Year		
2021	Ref	Ref
2022	1.122 (1.075–1.170)	0.970 (0.920–1.022)
2023	**1.305 (1.249–1.364)**	1.026 (0.972–1.083)
Delivery system redesign^e^		
No	Ref	Ref
Yes	**12.116 (10.851–13.527)**	**9.883 (8.739–11.177)**
Self-management support^e^		
No	Ref	Ref
Yes	**3.433 (3.097–3.805)**	**2.027 (1.761–2.334)**
Decision support^e^		
No	Ref	Ref
Yes	**1.093 (1.051–1.137)**	**0.791 (0.753–0.832)**
Clinical information systems^e^		
No	Ref	Ref
Yes	**7.007 (6.711–7.317)**	**7.738 (7.349–8.148)**
Healthcare organization^e^		
No	Ref	Ref
Yes	**1.208 (1.161–1.256)**	**1.165 (1.106–1.228)**
Community resources^e^		
No	Ref	Ref
Yes	**1.452 (1.282–1.644)**	1.152 (0.959–1.385)

Bold values indicate statistical significance at the *p* < 0.05 level

^a^Facility provides at least one medication for OUD treatment

^b^Odds ratio and 95% confidence intervals

^c^Adjusted odds ratio and 95% confidence intervals

^d^This includes Puerto Rico, the Commonwealth of the Northern Mariana Islands, the Republic of Palau, and the US Virgin Islands

^e^Element of the Chronic Care Model; construction of each element outlined in Supplemental Table 1

## Discussion

This national analysis of 2021–2023 N-SUMHSS data provides new evidence on the relationship between integrated treatment services for co-occurring mental health and substance use disorders and the provision of detoxification and MOUD across US treatment facilities. While integration was positively associated with both outcomes in substance use–focused facilities, no such benefit was observed in mental health–focused settings. These findings have several implications.

Results show that in facilities where substance use treatment is the primary focus, integrated services were associated with 26.5% higher odds of offering detoxification and 44.3% higher odds of offering MOUD. This pattern is consistent with prior empirical evidence indicating that integrated treatment for co-occurring disorders expands service breadth and improves coordination of care.^6,7,10^ For instance, OUD-specific evidence shows that linking withdrawal management/detox to the initiation and maintenance of MOUD improves outcomes and reduces relapse and acute care use, underscoring the importance of continuity from detox to maintenance.^16,17,21^ In addition, integrated facilities may have the staffing, protocols, and regulatory frameworks necessary to implement both behavioral and pharmacological interventions, which may be associated with increased treatment capacity for individuals with co-occurring disorders. For instance, the principal elements of SUD treatment programs include not only pharmacotherapies for addiction but also psychological interventions, behavioral therapies, and self-help groups.^33^ The association between integrated services and detoxification/MOUD provision may also reflect other shared underlying factors. For instance, larger, better resourced facilities that serve complex patient populations may be independently more likely to offer integrated services, detoxification, and MOUD.

In mental health–focused facilities, integration was associated with lower odds of detoxification availability and no significant difference in MOUD provision. This is consistent with evidence that many traditional mental health programs have limited SUD-specific capacity, stemming from gaps in cross-trained workforce, program infrastructure, and licensure/regulatory readiness, which constrains delivery of withdrawal management and MOUD.^23,34–36^ Accordingly, “integration” at the policy or administrative level may not translate into operational capability without targeted investment in staffing, training, clinical workflows, and financing, which is supported by literature reviews noting key barriers of OUD treatment.^35,36^ Mental health–focused facilities may also prioritize psychiatric stabilization and psychosocial therapies over pharmacological or withdrawal management approaches for SUD, reinforcing the need for dedicated SUD capacity to realize the benefits of integration. For example, an analysis of over 450 mental health treatment facilities across the USA found that only a third of community mental health facilities offered MOUD. Furthermore, that study showed that among facilities that do offer MOUD, the most common services offered to individuals with co-occurring mental health and substance use disorders include individual counseling (91%), group counseling (78%), telehealth for OUD (82%), and telehealth for MOUD (60%),^37^ highlighting limited application of integrative strategies in mental health facilities. Integrated services being associated with lower odds of detoxification availability may also reflect facilities with integrated services having stronger connections to external substance use treatment facilities than those that do not offer integrated services. However, due to the smaller sample size of mental health–focused facilities in the current study, further research would be needed to confirm these patterns.

Beyond integration, elements of the CCM, particularly delivery system redesign and clinical information systems, were strongly associated with detox and MOUD provision. Facilities with delivery system redesign had nearly 10 times the odds of providing MOUD compared to those without. This is consistent with CCM literature showing that structured, evidence-based workflows and robust information systems enable more reliable care delivery, and with SUD implementation studies demonstrating that organizational/process redesign and data systems increase access to MOUD.^12,23,38,39^ Investments in these elements may yield greater gains in treatment availability than integration alone, especially in facilities currently lacking operational capacity.^12,38,39^ Facility ownership may also play a significant role in whether facilities offer detox and MOUD.

In the adjusted models, private for-profit facilities had higher odds of offering detox but lower odds of offering MOUD than public facilities, and private nonprofits were less likely than public facilities to offer either service. These patterns are consistent with prior evidence that for-profit opioid treatment programs are less likely than nonprofit/public programs to offer comprehensive ancillary services, while more comprehensive MOUD offerings are associated with government or nonprofit ownership.^40–42^ For instance, past research found that OTPs offering three different types of MOUD treatment had significantly higher odds of being private nonprofit or government operated than private for profit.^41^ Financing and infrastructure factors, such as medical staffing, regulatory readiness, and fixed costs, also shape where medication and withdrawal management capacity can be built and sustained.^40–42^ For example, one study showed programs that relied on Medicaid funding were significantly more likely to implement MOUD, while programs that depended on criminal justice funding were associated with lower rates of MOUD implementation. ^43^

### Limitations

While this study provides important findings on the association between integrated care and the availability of detox and MOUD, several limitations should be noted. This study is cross-sectional and cannot establish causality between integration and service provision. Restricting the sample to facilities reporting any substance use services may overestimate integration prevalence. All measures are based on self-reported facility data, which may be subject to bias. For instance, facilities may misclassify services due to differences in interpretation of survey items or limited knowledge of services offered. Also, social desirability bias may influence facilities to overreport the availability of guideline-recommended services and underreport gaps in care. Even with including many relevant determinants of detox and MOUD, such as elements of the CCM, all selected measures were limited to those provided by N-SUMHSS, and proxy measures were used to reflect elements of the CCM (Supplemental Table 1). Such proxy measures may not fully capture the CCM elements. In addition, unobserved confounding may have occurred as important determinants of detox and MOUD, such as local policies, rurality, and stigma, are not included in N-SUMHSS. Generalizability may also be limited. Specifically, mental health–focused facilities had a smaller sample size than other primary treatment focuses (i.e., around 6% of the sample). As a result, the findings among this group should be interpreted with caution, and the findings may be more reflective of substance use–focused and mixed-focused facilities, potentially limiting the generalizability of these results when applied to mental health–focused settings. Despite these limitations, the large, nationally representative sample and adjustment for multiple facility-level factors strengthen the robustness of these findings that can guide optimal resource allocation and treatment offerings for policymakers and healthcare professionals.

## Conclusion

Integrated services for co-occurring disorders are associated with greater availability of detoxification and MOUD in substance use–focused treatment facilities but not in mental health–focused settings. Facility infrastructure and operational models are strong predictors of service availability. Tailoring integration strategies to the facility’s primary focus and enhancing core capacity elements, such as delivery system redesign, self-management support, and clinical information systems, may be linked with access to evidence-based treatments for individuals with co-occurring mental health and substance use disorders.

## Implications for Behavioral Health

These findings highlight that simply encouraging integration is insufficient to ensure equitable access to detoxification services and MOUD across all facility types. Policymakers and behavioral health system leaders should tailor strategies to facility focus: In substance use–focused facilities, expanding integrated services may be a high-yield approach to building detox and MOUD capacity, whereas in mental health–focused facilities, integration must be paired with targeted investment in workforce training, licensure expansion, and infrastructure to support SUD-specific services.^23,34,35^ Specifically, in substance use–focused facilities, expanded integrated services may include standardized mental health screening, referral networks, and on-location mental health professionals, supported by reimbursement models that incentivize integrated services. In mental health–specific facilities, developing SUD programs will require parallel expansion of grants, reimbursement policies, and prescribing policies that enable these facilities to deliver MOUD, detoxification services, and other SUD-specific care.

Geographic disparities observed in this data, consistent with prior work on rural access gaps, indicate the need for region-specific interventions, including telehealth expansion and targeted grant support.^24,44–46^ Telehealth services for MOUD and other healthcare needs among patients living in remote or underserved areas have helped reduce barriers to treatment.^47^ Federal grant funding has also supported expansion of MOUD by covering medication and treatment costs, particularly for individuals with financial constraints. However, sustained improvement depends not only on funding but also on coordination with pharmacies, education to address stigma and misconceptions around MOUD, and policies that support MOUD delivery in rural and underserved areas.^48^

Future behavioral health research should evaluate, using longitudinal designs, whether changes in integration status lead to subsequent increases in detox and MOUD availability. Qualitative studies could explore barriers and facilitators to implementing SUD-specific services in mental health–focused facilities and other low-capacity settings. Finally, linking N-SUMHSS to patient-level data would allow assessment of whether facility-level integration and Chronic Care Model elements translate into improved outcomes for people with co-occurring mental health and substance use disorders.

## Supplementary Information

Below is the link to the electronic supplementary material.

ESM 1 Supplementary file 1 (DOCX 18.7 KB)

## Data Availability

Data for this study were obtained from the 2021–2023 National Substance Use and Mental Health Services Survey (N-SUMHSS), a publicly available facility-level dataset administered by the Substance Abuse and Mental Health Services Administration (SAMHSA). These data can be accessed through the SAMHSA Data website (https://www.samhsa.gov/data/) in accordance with SAMHSA’s data use policies. No additional data beyond those provided in N-SUMHSS were collected or generated by the authors for this study.

## Declarations

### Reporting Guideline

STROBE (Strengthening the Reporting of Observational Studies in Epidemiology).
