# Supplementary material for: Association Between Integrated Treatment Services and Access to Detoxification and MOUD Across US Treatment Facilities
Source: J Behav Health Serv Res. 2026 Jun 8;53(4):574–89. doi: 10.1007/s11414-026-10003-9 (PMC13658384; doi:10.1007/s11414-026-10003-9)
Supplement: Supplementary file 1 [file 11414_2026_10003_MOESM1_ESM.docx]

| Supplemental Table 1. Elements of the chronic care model: application to detoxification and MOUD services and their proxy measure from NSUMHSS; adapted from Watkins et al. | | |
| --- | --- | --- |
| Element of the Chronic Care Model | **Application to detoxification and MOUD services** | **Proxy Measure from NSUMHSS** |
| Delivery system redesign | Is the facility designed to ensure evidence-based care for patients with OUD? | Facilities received a positive indication for delivery system redesign if they implement any of the following evidence-based strategies:   - Frequently uses 12-step facilitation for opioid use - Frequently uses brief intervention for opioid use - Frequently uses contingency management/motivational incentives for opioid use - Frequently uses trauma-related counseling for opioid use - Frequently uses anger management for opioid use - Frequently uses matrix model for opioid use |
| Self-management support | Are patients encouraged to become informed and competent self-managers of their OUD? | Facilities received a positive indication for self-management support if they provide patients with education about substance use and providing counseling to help them manage or overcome substance use issues. |
| Decision support | Is there expert consultation available to help clinicians adhere to evidence-based OUD treatment practices? | Facilities received a positive indication for decision support if they provide patients with a professional consultant who assists in managing substance use issues. |
| Clinical information systems | Do clinical information systems help providers plan and deliver care for both individuals and populations of patients with OUD and to monitor performance? | Facilities received a positive indication for clinical information systems if they implement a complete medical history and physical examination. |
| Health care organization | Is the leadership supportive, and are resources provided to support the delivery of OUD care? | Facilities received a positive indication for health care organization if they are licensed or certified by their state substance use agency. |
| Community resources | Are patients linked to community OUD recovery resources? | Facilities received a positive indication for community resources if they implemented any of the following recovery resources:   - Recovery mentoring/peer support - Recovery self-help groups - Recovery housing assist - Recovery employment - Recovery assistance social services - Recovery coach - Facility provides Case Management - Transitional discharge plan |

1.	Watkins KE, Hunter SB, Wenzel SL, et al. Prevalence and characteristics of clients with co-occurring disorders in outpatient substance abuse treatment. *Am J Drug Alcohol Abuse*. Nov 2004;30(4):749-64. doi:10.1081/ada-200037538
